# Ill-Defined Deaths in Slovenia: Implications for Mortality Data Quality and Burden of Disease Studies

**DOI:** 10.2478/sjph-2026-0020

**Published:** 2026-09-01

**Authors:** Tina Lesnik, Ivan Eržen, Lijana Zaletel Kragelj, Periklis Charalampous, Elena von der Lippe, Annelene Wengler, Brecht Devleesschauwer

**Affiliations:** National Institute of Public Health, Trubarjeva cesta 2, 1000 Ljubljana, Slovenia; University of Ljubljana, Medical Faculty, Department of Public Health, Zaloška cesta 4, 1000 Ljubljana, Slovenia; Department of Epidemiology and Public Health, Sciensano, Rue Ernest Blerot 1, 1070 Anderlecht, Belgium; Institute of Health and Society (IRSS), Université Catholique de Louvain, Brussels, Belgium; Department of Public Health, Erasmus MC, University Medical Center Rotterdam, Dr. Molewaterplein 40, 3015 GD Rotterdam, The Netherlands; Department of Epidemiology and Health Monitoring, Robert Koch Institute, Gerichtstraße 27, 13347 Berlin, Germany; Department of Translational Physiology, Infectiology and Public Health, Ghent University, Salisburylaan 133, 9820 Merelbeke, Belgium

**Keywords:** Cause of death, Mortality, Heart failure/epidemiology, Ill-defined causes of death, Age factors, Geographic variation/epidemiology, vzrok smrti, umrljivost, časovne serije, slabo opredeljeni vzroki smrti, starostna porazdelitev, razlike med, spoloma

## Abstract

**Introduction:**

Ill-defined deaths (IDDs) are deaths assigned non-specific or insufficiently informative causes that do not identify a distinct underlying disease or injury. They reduce the accuracy of mortality statistics. In Slovenia, the overall proportion, structure and distribution of IDDs have not yet been comprehensively evaluated. This study examined the most frequent IDDs and their demographic and regional patterns in Slovenia from 2010 to 2021.

**Methods:**

Individual-level mortality data from 2010 to 2021 were analysed. Deaths assigned ICD-10 codes denoting vague/non-specific conditions were classified as IDDs using the GBD 2019 cause list. Frequencies and distributions were examined overall and by sex, age group, and regions.

**Results:**

Of 243,166 deaths, 53,020 (21.8%) were IDDs. The proportion was higher in females and increased with age. Heart failure was the most frequent IDD and showed a distinctly higher share among females. Other common IDDs included unspecified stroke, unspecified lower respiratory infections, unspecified cancers, and R99-coded (Ill-defined and unknown cause of mortality) deaths. Clear variation in age groups was observed: poisoning-related IDDs were concentrated in young adults, R99 predominated in working age groups, and cardiovascular-related IDDs dominated in older adults. Regional distributions of the top categories of IDDs resembled the national one, except for unspecified cardiomyopathy, which appeared prominently in two north-eastern regions.

**Conclusions:**

Heart failure is the major contributor to IDDs in Slovenia, with demographic and regional variation. The findings highlight the need to strengthen death certification and improve the quality of mortality data to support burden-of-disease analyses.

## Izvleček

**Uvod:**

Slabo opredeljeni vzroki smrti (angl. ill-defined deaths, IDD), v okviru metodologije Global Burden of Disease pogosto označeni tudi kot »garbage codes«, vplivajo na interpretacijo statistike umrljivosti in ocen bremena bolezni. Strukturne značilnosti IDD v Sloveniji smo že opisali, namen te raziskave je bil podrobneje analizirati njihove časovne trende.

**Metode:**

V raziskavo so bile vključene vse registrirane smrti v Sloveniji v obdobju 2010–2021 na podlagi Zbirke podatkov o umrli osebi. Izračunani so bili letni grobi deleži skupnih IDD in petih najpogostejših kategorij slabo opredeljenih vzrokov smrti (CoIDD) v celotni populaciji in po spolu. Za oceno vpliva starostne strukture je bila uporabljena neposredna starostna standardizacija na starostno porazdelitev vseh smrti v letu 2010 kot skupni izhodiščni standard. Časovni trendi starostno standardiziranih deležev skupnih IDD so bili dodatno analizirani z Joinpoint regresijo.

**Rezultati:**

V obdobju 2010–2021 je bilo med 243.167 smrtmi v Sloveniji 53.021 (21,8 %) takih, ki so bile razvrščene med IDD. Grobi delež IDD se je zmanjšal s 24,5 % v letu 2010 na 15,9 % v letu 2021, starostno standardizirani delež pa s 24,5 % na 14,3 %. Joinpoint analiza je pokazala prelom okoli leta 2016, pri čemer pred tem letom trend ni bil statistično značilen, po njem pa je sledil izrazit upad. Starostno standardizirani deleži IDD so bili v celotnem obdobju višji pri ženskah kot pri moških, vendar so bili časovni trendi po spolu podobni. Trendni vzorci po posameznih kategorijah CoIDD so bili heterogeni: srčno popuščanje je ostalo najpogostejša kategorija; njegov trend je bil nelinearen, z začetnim porastom in poznejšim strmim upadom. Neopredeljena možganska kap in neopredeljene okužbe spodnjih dihal so izrazito upadle, neopredeljeni raki so ostali razmeroma stabilni, kategorija R99 pa se je povečevala.

**Zaključki:**

Celovito razumevanje časovnih trendov slabo opredeljene umrljivosti v Sloveniji zahteva sočasno obravnavo skupnega deleža IDD, analizo po posameznih kategorijah in spolu ter vplivu starostne strukture smrti. Takšen pristop pokaže, da IDD niso homogena skupina, temveč dinamični in heterogeni sestavni del podatkov o umrljivosti. Ugotovitve prispevajo k boljši interpretaciji statistike umrljivosti in predstavljajo pomembno osnovo za nadaljnje analitične postopke, kot je redistribucija vzrokov smrti v raziskavah bremena bolezni.

## 1 INTRODUCTION

Routine health data are important for monitoring population health, evaluating healthcare interventions, and informing policy decisions. They provide timely, continuous, and cost-effective information; however, data quality—often affected by incomplete reporting, data entry errors, and lack of standardisation—can limit their validity and reliability, especially for in-depth analyses (1). Mortality and causes-of-death data are key elements of routine data registration systems. Accurately determining and coding the underlying causes of death (CoD) is critical for producing meaningful mortality statistics. To ensure reliable and comparable data, CoD data are recorded using the International Classification of Diseases (ICD) system of the World Health Organisation (WHO) (currently 10th revision: ICD-10). With ICD revisions, the number of codes describing symptoms, signs, or ill-defined conditions increased. Despite WHO recommendations to record the underlying CoD as the event that initiated the chain of pathological processes resulting in death (2), not all coded “underlying” CoD fulfil these criteria and are suitable for burden-of-disease estimates.

Such codes are commonly referred to as ill-defined deaths (IDDs) when they are recorded as the underlying cause of death. They include ICD-10 codes that are too vague/non-specific to identify a distinct disease or injury (2, 3). In the Global Burden of Disease (GBD) framework, these codes are referred to as garbage codes (4). Besides the completeness of death registration, the proportions of IDDs are an important measure of the quality of the mortality data. A high proportion of IDDs is generally considered an indicator of limitations in death certification practices and may reduce the accuracy of mortality statistics used for public health decision-making (5).

Increasing attention is being paid to this field since the establishment of the GBD study (6, 7). Within its framework, a thorough list of IDD categories was developed and updated with GBD study cycles. The current list is comprehensive, including more than 5,000 four-digit ICD codes (8). However, in its current form, the classification has been developed for use within the GBD study. Alongside the GBD study, several national burden of disease studies have been conducted across Europe, including Germany, Scotland, the Netherlands, Belgium and France (3, 9,10,11,12). These studies commonly draw on the GBD framework, while adapting selected methodological elements to national data sources, analytical priorities and public health needs.

Slovenia has a well-developed system of routine health data and has recently also begun using them for more in-depth analyses. Regarding mortality data, international analyses indicate that the proportion of IDDs in Slovenia is not particularly high, amounting to roughly one-fifth of all deaths (8). Nevertheless, further improvements in mortality data quality remain possible, particularly in accurately determining the underlying cause of death.

To support such improvements, this study represents a first step in systematically characterising national IDDs patterns during the period 2010–2021. Specifically, the objectives were to determine the proportion of IDDs, identify the most frequent IDD categories, and examine their distribution across population subgroups according to sex, age and region.

## 2 MATERIALS AND METHODS

### 2.1 Study design

This study was designed as a routine individual-level data study. Nationally representative CoD data from 2010 to 2021 were pooled and analysed together.

### 2.2 Data sources

Routine mortality data were obtained from the Medical Report on the Deceased database of the National Institute of Public Health in Slovenia (NIPH), which compiles death certificates from 58 administrative units nationwide (13). NIPH coders centrally review and validate the primary underlying CoD, conducting investigations to resolve ambiguities. CoD are coded according to WHO ICD-10 rules (2, 14). Population data were obtained from the Statistical Office of Slovenia (15).

### 2.3 Observed outcome

All IDDs not clearly indicating distinct diseases/injuries or denoting symptoms, signs, or temporary conditions (e.g. ICD code I50.9: Heart failure, unspecified), were considered as the observed outcome. This also includes IDDs with an ICD code denoting no information on the CoD at all. (R99: Other ill-defined and unspecified causes of mortality). The definition of IDDs was based on the GBD 2019 cause list (16), which includes a category referred to as garbage codes; in the present study, this category is termed IDDs. ICD codes included in the GBD 2019 IDDs list were systematically reviewed and compared with the IDDs list used in previous GBD study cycles. Public health researchers and epidemiologists with expertise in chronic non-communicable diseases conducted the review. Codes were retained as valid causes of death when they represented epidemiologically meaningful disease entities in the Slovenian context, particularly for diabetes mellitus and alcohol-related diseases, which are relevant to national public health monitoring and prevention programmes.

As a result of this assessment, a total of 2,274 ICD codes were excluded from the IDDs definition. A sensitivity analysis was conducted to assess the impact of this modification on the proportion of IDDs and the distribution of IDD categories.

The excluded ICD codes were alcohol-related liver disease (K70.4, K70.9), diabetes mellitus codes (E14.0–E14.8), selected haematological malignancies (C91.1, C91.4, C91.5, C91.7), substance use–related disorders (F19.1, F19.2, F19.5), and peripheral arterial embolism and thrombosis (I74.0–I74.9).

The resulting group of IDDs was subsequently mapped to IDD categories based on the GBD 2021 IDD category list (8), which provides a standardised grouping of ill-defined causes. Minor adaptations were made to facilitate the analysis. Specifically, the categories “Left heart failure” and “Heart failure, unspecified right or left” were merged into a single category, “Heart failure, left or unspecified”. In addition, the category “Unspecified site cancer” was combined with other site-specific unspecified cancer categories (e.g. unspecified respiratory cancer), resulting in a unified category, “All unspecified cancers”. Within the category “All ill-defined causes of death”, ICD code R99 accounted for approximately 95.1% of all deaths. Because of this strong predominance, R99 was analysed separately.

The IDDs included in the analysis and their corresponding IDD categories are presented in Table 1. As the number of IDD categories was substantial and many accounted for only very small proportions, results are reported by presenting the five most frequent IDD categories, with the remaining categories aggregated into a single category labelled “Other”.

**Table 1:** Mapping of ICD-10 codes to IDD categories used in the analysis.

**IDDs group**	**ICD codes**
Heart failure, left-sided or unspecified	I50, I50.0–I50.9, J81
Unspecified type of stroke	I64, I67, I67.8–I68, I68.8–I69, I69.4–I69.8
Unspecified lower respiratory infections	J15, J15.9, J17–J19.6, J22-J22.9, P23, P23.5–P23.9
All unspecified cancers	C1, C2, C3, C4, C5, C6, C7, C14-C14.9, C22, C22.9, C26, C35–C36, C39–C39.9, C42, C55–C55.9, C57, C57.9, C59, C68, C68.9, C75, C75.9, C76, C76–C76.4, C76.7-C77, C77-C77.9, C78-C79.1, C79, C79.2, C80.9, C87, C97, D00, D01, D01.4–D01.9, D02, D02.4–D02.9, D4, D07, D07.3, D08-D09, D09.1, D09.7, D09.9, D10, D10.9, D13, D13.9, D14, D14.4, D17–D21.9, D28, D28.9, D30, D30.9, D36–D36.0, D36.9, D37–D37.0, D37.6–D37.9, D38, D38.6, D39–D39.0, D39.7, D39.9, D41, D41.9, D44, D44.9, D48, D48.7–D49, D49–D49.0, D49.1, D49.7, D49.8–D49.9, D54, E34–E34.0, N84, N84.2–N84.8
Unspecified cardiomyopathy	I42–I42.0, I42.9
Shock, coma, asphyxia or convulsion	I46–I46.9, I95–I95.1, I95.8–I95.9, R03, R03.1, R09–R09.0, R09.2, R09.8, R40-R40.4, R55, R56, R56.1-R57.9
Central nervous system (CNS) intermediate causes	G91–G91.2, G91.4–G93, G93.1–G93.2, G93.4–G93.6, G94-G94.8
Poisonings by multiple or unspecified drugs, UDI	F19–F19.9, X40–X44.9, X46–X46.9, X49–X49.9, X55–X55
Poisonings by narcotics and psychodysleptics, UDI	Y12–Y12.9
Ill-defined and unknown cause of mortality	R99
Other IDDs	All remaining IDDs based on the GBD list not explicitly listed above (8)

### 2.4 Observed population subgroups

The outcome was observed in prespecified population subgroups by sex (male, female), age (age groups: < 20, 20–29, 30–39, 40–49, 50–59, 60–69, 70–79, 80–89, 90+), and twelve regions of Slovenia (Obalno-kraška, Goriška, Primorsko-notranjska, Gorenjska, Osrednjeslovenska, Posavska, Zasavska, Savinjska, Koroška, Pomurska, Podravska regions and Jugovzhodna Slovenija region).

### 2.5 Statistical analysis

The frequencies of IDDs among the deceased population, expressed as percentages, were calculated for the total population and the defined subgroups. The analysis was descriptive. Differences in the distribution of IDDs across sex, age groups and regions were assessed by comparing proportions. Data analyses were performed using R (version 4.3.1) (17), and QGIS (18) was used to create the choropleth map.

### 2.6 Ethical considerations

The analysis was based on anonymised national mortality data. Data use complied with the National Statistics Act, and no individual informed consent was required.

## 3 RESULTS

### 3.1 Description of the observed group

Over the 12-year study period, a total of 243,166 deaths were recorded in Slovenia, of which 53,020 were classified as IDDs according to the study-specific IDDs definition, corresponding to 21.8%. The distribution of IDDs proportions differed by sex and age group and is presented in Table 2. The sensitivity analysis showed that applying the unmodified GBD definition increased the overall proportion of IDDs from 21.8% to 22.7%. The additional deaths were distributed across the excluded diagnostic groups, mainly diabetes mellitus, alcohol-related liver disease and selected haematological malignancies. The ranking of the leading IDD categories remained unchanged, and no meaningful changes were observed in the main sex-, ageor region-specific patterns.

**Table 2:** Proportion of IDDs by sex and age group, Slovenia, pooled 2010–2021.

**Category**	**% of IDDs**
**Sex**	
Males	17.0
Females	26.5
**Age groups**	
< 20	10.1
20–29	18.9
30–39	18.3
40–49	11.4
50–59	9.4
60–69	10.6
70–79	15.2
80–89	26.3
90+	40.0

**Figure 1:**
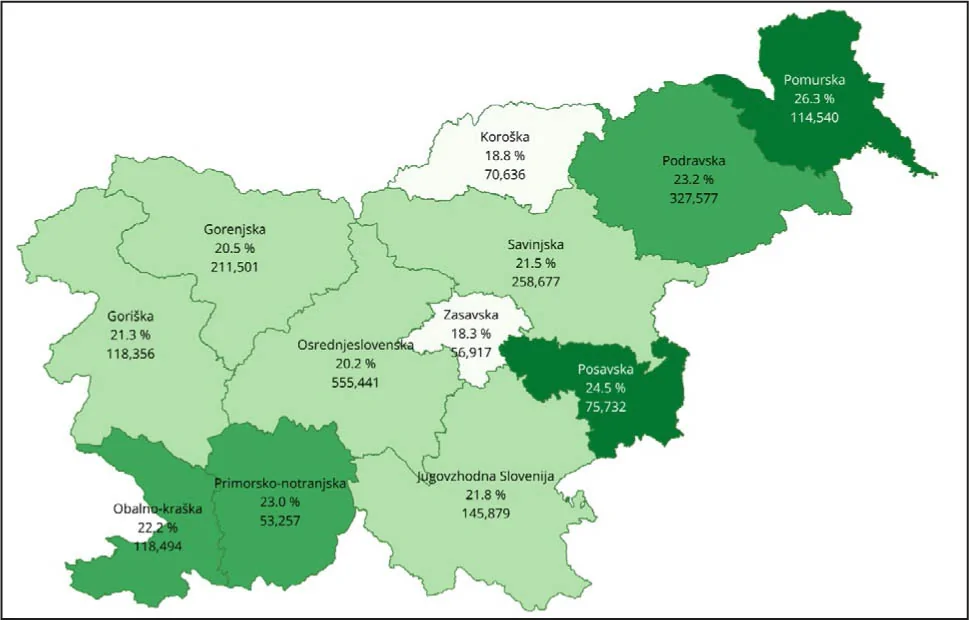
Proportion of IDDs by regions and population size, Slovenia, 2010–2021.

Regionally, the share of IDDs was lowest in the Zasavska region (18.3%) and highest in the Pomurska region (26.3%), with an absolute difference of 8 percentage points. In the remaining regions, the share of IDDs ranged from 18.8% (Koroška) to 24.5% (Posavska) (Figure 1).

### 3.2 Analysis of the distribution of IDD categories

#### 3.2.1 Distribution of IDDs in the total observed group

**Figure 2:**
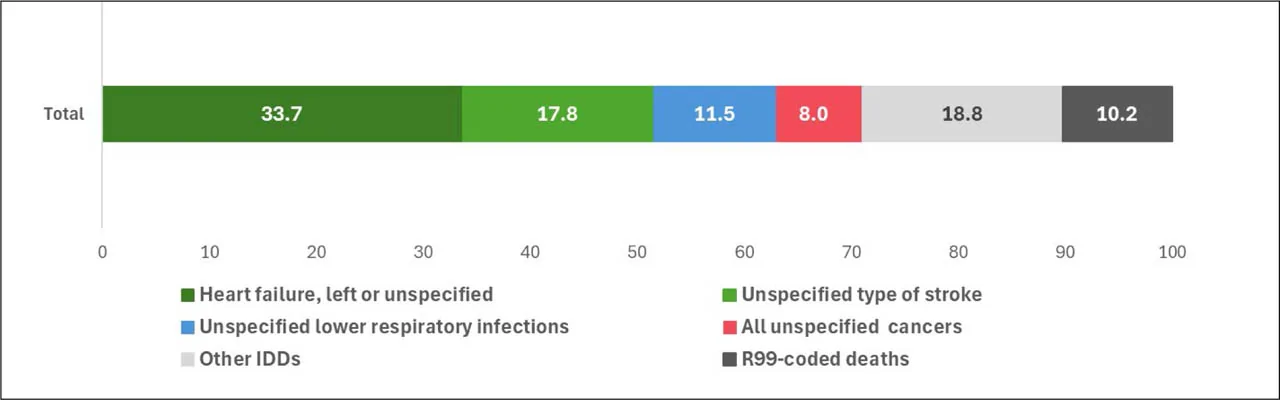
Most common causes of ill-defined deaths (as % of all ill-defined deaths) in the total population, Slovenia, 2010–2021. Sensitivity analysis indicated no change in the ranking of IDDs when the unmodified GBD IDDs definition was applied.

The most common IDD categories were heart failure, left-sided or unspecified (33.7%), followed by unspecified type of stroke (17.8%), and unspecified lower respiratory infections (11.5%), Figure 2.

#### 3.2.2 Distribution of IDDs by sex

**Figure 3:**
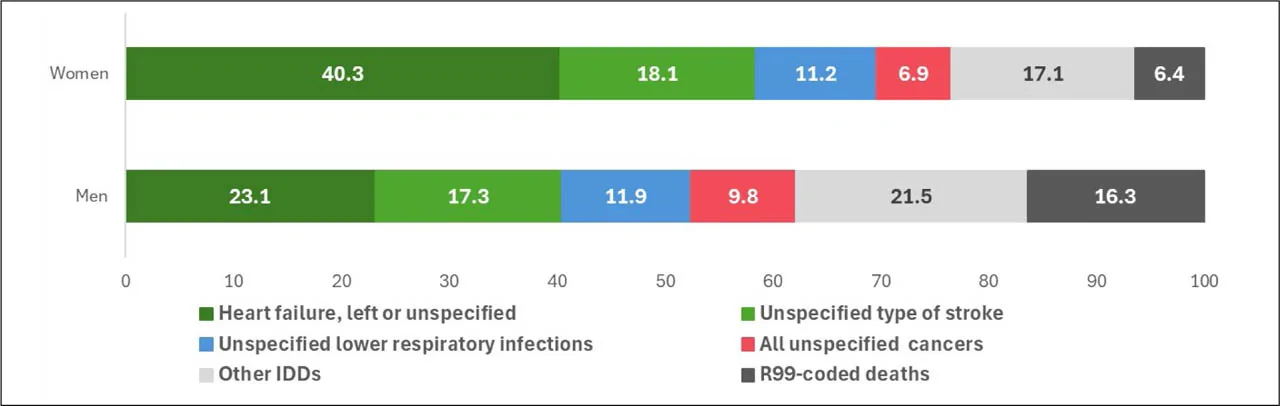
Percentages of most common ill-defined deaths (IDDs) by sex, Slovenia, 2010–2021.

The distribution and ranking of IDDs differed significantly between males and females. The most notable difference was observed in heart failure, where the proportion among females (40.3%) exceeded that among males (23.1%) (Figure 3). Conversely, males exhibited a higher proportion of R99-coded deaths, 16.3% vs. 6.4% in females (Figure 3).

Cancers with unspecified site ranked sixth among males and fifth among females, with proportions differing between the sexes (Figure 3). In contrast, unspecified types of stroke and unspecified lower respiratory infections showed only marginal differences. The category of other IDDs ranked second among males, while unspecified stroke and other IDDs were the second and third most frequent causes among females, respectively. Males had a substantially higher proportion of deaths classified as Other IDDs than females (21.5% vs. 17.1%) (Figure 3).

#### 3.2.3 Distribution of IDDs by age

**Figure 4:**
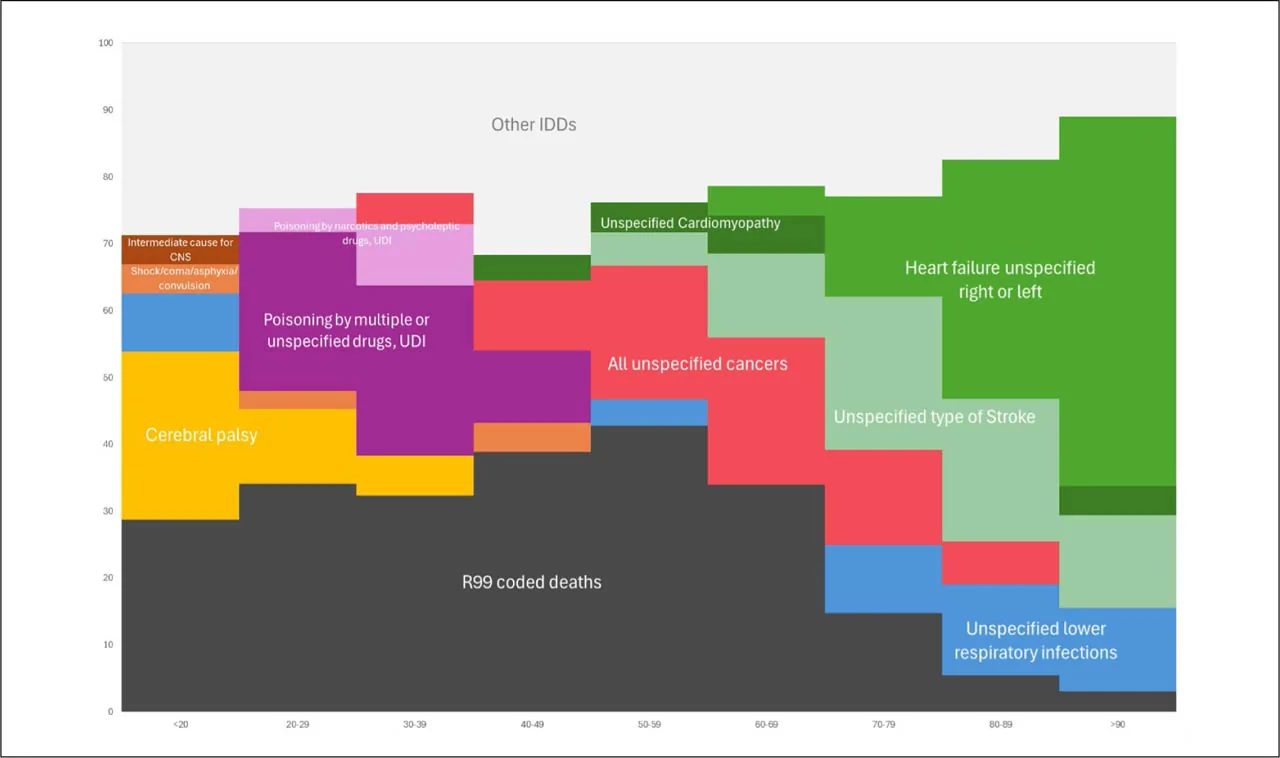
Percentage distribution of the five most frequent IDD categories by age group, Slovenia, 2010–2021.

The distribution of the most common IDDs varied across age groups (Figure 4). R99-coded deaths were the most consistently represented IDDs across all age groups, with the highest frequency in the fourth and fifth decades. Heart failure was overrepresented in the oldest age groups, while unspecified strokes showed moderate and relatively constant shares from the fifties onward. Unspecified cancers were distributed across many age groups, starting in the third decade and peaking in the fifth. Unspecified lower respiratory infections were present at both ends of the age spectrum, particularly among the youngest and oldest individuals (Figure 4). Poisonings by multiple or unspecified drugs, narcotics, and psychodysleptics of undetermined intent occurred predominantly in younger age groups. Deaths coded as cerebral palsy were observed mainly in the first four decades, with the highest share in the youngest age group (Figure 4).

#### 3.2.4 Distribution of IDDs by region

**Figure 5:**
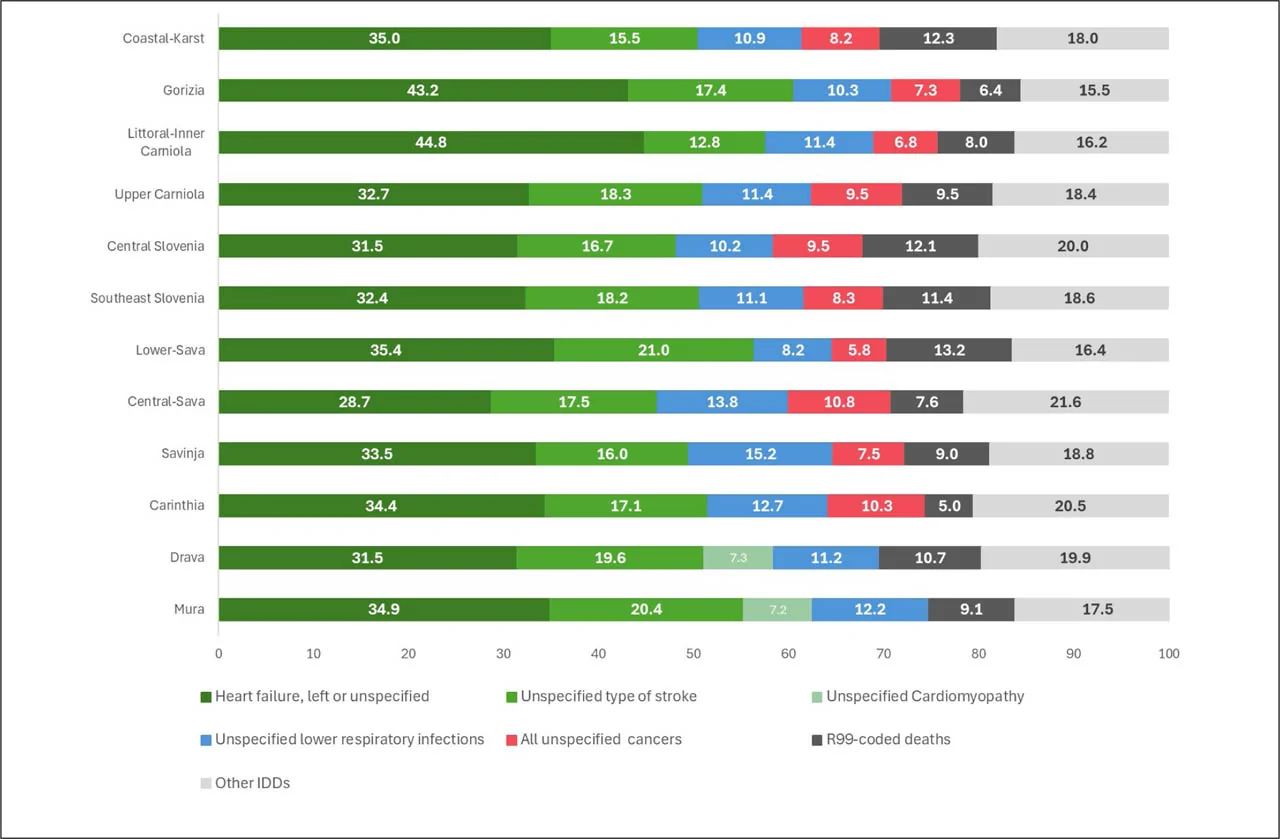
Percentage distribution of the most frequent IDD categories by region, Slovenia, 2010–2021.

Figure 5 displays the proportional distribution of the five most common IDD categories across Slovenia's twelve statistical regions, revealing substantial variation in cause composition.

Heart failure, left or unspecified, was the leading IDD in all regions, with the highest share recorded in Primorskonotranjska (44.8%) and Goriška (43.2%), and the lowest in Zasavska (28.7%). Unspecified type of stroke was generally ranked second, particularly in Pomurska, Posavska, and Podravska regions; however, in some regions, such as Savinjska and Osrednjeslovenska, its proportion was slightly lower and closely followed by other causes. Unspecified lower respiratory infections ranked among the top three causes in most regions, with the highest shares in the Savinjska, Pomurska, and Koroška regions. Notably, unspecified cardiomyopathy occurred only in Pomurska and Podravska, accounting for 7.2% and 7.3% of all IDDs, respectively, whereas all unspecified cancers were more prevalent in Zasavska, Gorenjska, and Obalno-kraška regions (Figure 5).

R99-coded deaths were most prominent in Posavska, Osrednjeslovenska, and Obalno-kraška, but were less so in Koroška and Primorsko-notranjska. The residual category, Other IDDs comprising less frequent causes, accounted for 15.5–26.2% of IDDs across regions, with the highest contribution observed in the Zasavska region and the lowest in the Goriška region.

## 4 DISCUSSION

### 4.1 Main findings

Our study provides the first comprehensive analysis of the proportions, structures, and distributions of ill-defined deaths in Slovenia. The most frequently coded IDDs in Slovenia are heart failure. Excluding R99, which provides no information on the underlying cause, the next most frequent are unspecified stroke, lower respiratory infections and unspecified cancers.

In Slovenia, overall mortality is characterised by a high proportion of cardiovascular deaths, particularly at older ages and among females (19). Females in Slovenia live longer, with a life expectancy approximately six years higher than that of males (15). In line with the general mortality profile, IDDs are more frequent among older individuals and females, with heart failure being particularly prominent among females. Heart failure is the leading category across all regions and represents the main contributor to IDDs, making it a key indicator of mortality data quality.

Although cardiovascular disease IDDs are most frequently reported in other studies (20, 21), and heart failure ranks among the leading IDDs in several national analyses (9, 22, 23), such a pronounced predominance of heart failure—particularly among females—has not been observed in other countries to the extent seen in Slovenia. Notably, this is in line with the Johnson et al. study, which analysed IDDs using GBD data, and ranked heart failure as the leading IDD for Slovenia (35.5%) (8).

Heart failure is classified as a circulatory disease, but it represents a final clinical condition that can result from many different underlying causes. As such, it represents a key ill-defined category, as the underlying cause may stem from non-communicable, communicable diseases, or injuries, and should be addressed in the redistribution process (21).

Cancers, the second leading cause of death in Slovenia (19), ranked fifth, indicating a smaller role in IDDs compared with their overall contribution to mortality. This may reflect the clinical and diagnostic characteristics of cancer, as assignment of the underlying cause of death is usually straightforward when the cancer site and type are known. Ill-defined cancer-related deaths may therefore occur mainly when key diagnostic information is missing, such as tumour localisation or histological confirmation, potentially due to delayed diagnosis, rapid disease progression, or limitations in death certification or coding. The relative contribution of these mechanisms cannot be assessed using the available data.

Unspecified stroke represents a particular form of IDDs, as it may reflect either an ischaemic or haemorrhagic underlying mechanism. Recording stroke as unspecified may indicate that death occurred before diagnostic clarification was made, leaving the underlying cause undefined. This could be related to a rapid clinical course and to decisions not to pursue further diagnostic clarification, in which additional diagnostic information may not be considered clinically meaningful.

Non-specific lower respiratory infections, most often pneumonia, rank high among IDDs within the respiratory disease group. This IDDs group may arise when physicians completing the medical certificate of death do not pursue a more specific underlying cause or lack access to detailed diagnostic information, particularly when pathogen-specific identification is not available or not considered clinically relevant for treatment.

A considerable share of IDDs consists of R99 codes, with a markedly higher proportion among males. In Slovenia, these codes are used almost exclusively for deaths occurring abroad, as other situations with completely missing cause-of-death information are extremely rare. If the underlying causes of R99 deaths were known, their distribution would likely differ from that observed in the general population. Given the male excess in transport-related mortality in the general population (19), these causes may also be overrepresented among R99 deaths; however, this remains speculative due to the lack of information on underlying causes.

Regional differences in the proportion of IDDs were observed across Slovenia; however, they were modest, spanning approximately eight percentage points. The highest proportion was recorded in the Pomurje region, which is characterised by the oldest population structure (15) and less favourable mortality, morbidity, risk factor and socio-economic profiles (24, 25). As the regional comparisons are not age-standardised, part of the observed variation in IDD percentages is likely related to demographic differences rather than differences in death certification quality.

Regional variation in IDDs has also been reported in other European countries. Studies from Scotland, France and Germany identified regional differences in the use of IDDs and highlighted the importance of both population characteristics and certification practices when interpreting such patterns (3, 12, 26). While regional differences were also observed in Slovenia, the magnitude of variation was relatively modest, suggesting a comparatively homogeneous national mortality coding system. Although the overall distribution of IDDs was broadly similar across regions, differences in cause composition were evident. Unspecified cardiomyopathy ranked among the five leading IDDs only in the Podravska and Pomurje regions. Given the centralised coding of underlying causes of death, these differences are unlikely to reflect coding variability and may instead point to regional differences in death certification.

Regions with lower IDDs proportions, such as Zasavska or Koroška, showed a more heterogeneous IDDs composition, reflected in a higher contribution of the residual category Other IDDs, whereas regions with higher IDDs proportions exhibited greater concentration of deaths within a limited number of leading IDDs. As R99-coded deaths in Slovenia predominantly reflect deaths abroad, regional variation in R99 likely reflects differences in population mobility and administrative data flows rather than coding practices.

The distribution of IDDs varies across age groups. R99 consistently ranks among the five most common IDDs in all age groups, with particularly high proportions in the working-age population. As the R99 code in Slovenia is almost exclusively used for deaths abroad, while its use for deaths within the country is extremely rare (according to personal correspondence with national cause-of-death coders), this subgroup may exhibit a distinct cause-of-death pattern. In older age groups, heart failure and unspecified stroke predominate, consistent with established mortality patterns in ageing populations (3, 7).

In contrast, the distribution in younger and working-age groups reveals a more diverse picture. Cerebral palsy appears frequently as an IDD in childhood, which is expected from a clinical perspective. Non-specific cancers appear among IDDs in the working-age population. Although deaths in this age range are relatively few, cancers in general represent an important CoD, and the use of non-specific cancer codes may reflect incomplete diagnostic information, limited access to medical records, or rapid disease progression; however, these explanations cannot be evaluated using the available data. Another noticeable peak in the IDDs' share is observed in young adulthood. In this age group, the most common IDDs are poisonings with undetermined intent, typically involving narcotics, psycholeptic drugs, or various unspecified substances. These deaths likely represent intentional self-harm or unintentional overdoses. This finding aligns with established CoD patterns in this age group, where drug-related deaths and intentional self-harm contribute significantly to mortality (23).

### 4.2 Strengths and limitations

This study has several strengths. It is based on national mortality data processed through a single, centralised coding system, which reduces potential bias arising from regional differences in coding practices, as may occur in countries with decentralised coding institutions. As a result, determination of the underlying cause of death is not subject to regional coding variability. In the absence of automated coding tools such as IRIS (27), all death certificates are manually reviewed, with additional investigation undertaken when necessary. This centralised and manual system ensures a high level of consistency and oversight.

The study also has some limitations. First, the electronic mortality database currently records only the underlying cause of death and does not include an electronic record of contributing conditions and comorbidities. However, this coding practice is well established and aligned with WHO guidelines for mortality statistics. The planned implementation of an electronic death certification system is expected to enable systematic recording of the full sequence of CoD.

Second, within the GBD framework, the list of IDDs has expanded over successive study cycles. We assessed the newly added IDDs and retained some of them as specific, non-IDD causes where this was considered more appropriate in the Slovenian context, as described in the Methods. These codes occurred rarely and had a negligible impact on overall IDDs proportions. While this reduced direct comparability with the latest GBD classification, it did not affect the results presented in this study.

Finally, we did not adjust the results for potential confounding factors, such as age, and therefore did not age-standardise comparisons between sexes and regions. The present analysis aimed to describe the magnitude and structure of IDDs in an unadjusted form, providing a baseline for future analyses.

Building on these findings, future research will examine temporal trends in IDDs and assess approaches to redistribute them to specific underlying causes of death. In addition, the planned implementation of an electronic death certification system may further enhance data completeness by enabling systematic recording of the full sequence of CoD. Together with continued guidance for physicians completing death certificates, these measures could reduce the use of non-specific underlying causes and improve the utility of mortality data.

## 5 CONCLUSIONS

This study provides the first comprehensive overview of ill-defined deaths in Slovenia. More than one-fifth of deaths were classified as IDDs, with variation by sex, age and region. Heart failure was the predominant IDD category, particularly among females and older age groups.

The findings identify key areas for improving mortality data quality, particularly the certification of heart failure, unspecified stroke, lower respiratory infections and R99-coded deaths as underlying causes of death. They also provide a baseline for future analyses of temporal trends and the redistribution of IDDs within Slovenian burden-of-disease studies.
